# ﻿Three new species of *Agaricus* (Agaricaceae, Agaricales) from southern China

**DOI:** 10.3897/mycokeys.119.154278

**Published:** 2025-06-25

**Authors:** Shi-en Wang, Si-ang Chen, Hai-chen Huang, Dong-mei Lin, Peng-hu Liu

**Affiliations:** 1 National Engineering Research Center of Juncao Technology, College of JunCao Science and Ecology, Fujian Agriculture and Forestry University, Fuzhou, China Fujian Agriculture and Forestry University Fuzhou China; 2 College of Agriculture, Fujian Agriculture and Forestry University, Fuzhou, China Fujian Agriculture and Forestry University Fuzhou China; 3 College of Life Sciences, Fujian Agriculture and Forestry University, Fuzhou, China Fujian Agriculture and Forestry University Fuzhou China

**Keywords:** *Agaricus*, morphology, multi-locus phylogeny, new species, taxonomy

## Abstract

The genus *Agaricus* is the most species-rich genus within the Agaricaceae, comprising more than 500 species globally. Here, we describe three new species of *Agaricus* based on morphological and multi-locus (ITS + nrLSU + *tef1-α*) phylogenetic analyses: *Agaricusaurantifibrillosus*, *A.fafuinus* and *A.shenzhenensis*. *Agaricusaurantifibrillosus* and *A.fafuinus* were collected at Fujian province, while *A.shenzhenensis* was discovered in Shenzhen City, Guangdong Province. The morphological descriptions of each new species are accompanied by basidiomata photographs, and illustrations of microscopic structures.

## ﻿Introduction

*Agaricus* L. is the type genus of the family Agaricaceae Chevall. (Donk 1962; Parra 2008; Kerrigan 2016; Zhao et al. 2016; Bau 2018). Species within *Agaricus* are primarily saprophytic fungi, typically inhabiting forest ecosystems or grasslands (Zhao et al. 2011; Chen et al. 2017). However, recent studies have expanded their known ecological niches, with documentation of lignicolous taxa such as *A.subiculosus* Miller, Angelini, L.A. Parra & Linda J. Chen growing on decaying wood substrates (Parra et al. 2024a). Although a minority of *Agaricus* species are toxic and may provoke gastrointestinal distress, the majority exhibit significant edible and medicinal value (Wu et al. 2019; Boxshall et al. 2021; Jaichaliaw et al. 2021; He et al. 2022). Currently, several species within *Agaricus* are utilized for food and nutraceutical applications, including *A.bisporus* (J.E. Lange) Imbach, *A.flocculosipes* R.L. Zhao, Desjardin, Guinb. & K.D. Hyde and *A.subrufescens* Peck (Wisitrassameewong et al. 2012; Zhao et al. 2012, 2016).

As outlined in our previous studies (Wang 2024; Wang and Bau 2024), the taxonomy of *Agaricus* has experienced substantial development over the past two decades, due to the incorporation of molecular characters and phylogenetic methodologies to the traditional morphological characters. To date, *Agaricus* comprises six subgenera, 27 sections (Zhao et al. 2016; Chen et al. 2017; Callac and Chen 2018; He et al. 2018a; Parra et al. 2018a; Ortiz-Santana et al. 2021; Hussain et al. 2022). This study focuses on three subgenera and three sections within *Agaricus*, viz., A.sect.Agaricus in A.subg.Agaricus, A.sect.Catenulati in A.subg.Pseudochitonia, and A.sect.Minores in A.subg.Minores.

The type species of A.sect.Agaricus is *A.campestris* L., which shows the following taxonomic characteristics: pileus surface unchanging or slightly yellowing, rarely rufescent on touching; context often turning pink or strongly reddening; odor usually indistinct or mushroomy, sometimes anise; annulus superous or intermediate, simple or double, membranous or fibrillose; KOH and Schäffer’s reactions negative on white areas of the surface of the pileus; cheilocystidia absent or indistinct, basidia-like, some species abundant, large, globose, piriform or ovoid, never catenulate (Zhao et al. 2016).

The type species of A.sect.Catenulati is *A.arabiensis* S. Hussain & Al-Sadi. Species of the section are characterized by: medium-sized basidiomata; white context unchanging when bruised or handled; a phenolic odor; a slightly yellow KOH reaction and negative Schäffer’s reactions; cheilocystidia globose to subglobose or broadly ellipsoid, the anteterminal elements subglobose or cylinderical, forming a chain-shaped structure (Hussain et al. 2022).

Agaricussect.Minores with the type species *A.comtulus* Fr.. This section has the following phenotypic characters: basidiomes often slender, small-tomedium sized; surface of pileus often discoloring yellow on touching; context often turning yellow on exposure; odor of anise or bitter almonds; annulus superous, simple, thin, fragile; KOH and Schäffer’s reactions positive; cheilocystidia simple, clavate, pyriform, sometimes absent, scattered or rare (Zhao et al. 2016).

The genus *Agaricus* has attracted increasing attention from taxonomists in recent years. In 2024, 26 novel species were described globally (Arya and Pradeep 2024; Crous et al. 2024; Bashir et al. 2024; Guzmán-Guillermo et al. 2024; Liang et al. 2024; Liu et al. 2024; Nawaz et al. 2024; Palestina-Villa et al. 2024; Parra et al. 2024a, 2024b; Ullah et al. 2024) following our previous publication on 11 January (Wang and Bau 2024). Notably, *A.baiyunensis*, *A.cacainus* and *A.praeclarefibrillosus* were discovered in southern China (Liang et al. 2024). Additionally, *A.totalaiiensis* M. Ishaq, M. Fiaz & A.N. Khalid is currently under review (https://preprints.arphahub.com/article/133080/, accessed on 4 March 2025).

In this study, we further expand the known diversity of *Agaricus* in China by describing three new species based on morphological characteristics and molecular phylogenetic analyses, i.e., *A.aurantifibrillosus*, *A.fafuinus* and *A.shenzhenensis*.

## ﻿Materials and methods

### ﻿Morphological studies

The macro-morphological characteristics of the species were described using notes and photographs taken from fresh basidiomata collected during field collecting. The color description of the fresh basidiomata follows the Methuen Handbook of Color (Kornerup and Wanscher 1978). Microstructures were observed using a Nikon differential interference contrast (DIC) optical microscope (Nikon Corporation, Japan). Basidiospore descriptions followed the methodologies outlined in previous studies (Wang and Bau 2024). The symbol “(a) b–c (d)” is used to describe the size of basidiospores, where the “b–c” range represents 90% of the measured values, while the “a” and “d” are extreme values. “[Xav = e × f]” indicates the average size of basidiospores. “Q” refers to the ratio of length to width of a single basidiospore from the side view, and “Qav” refers to the average value of “Q” of all specimens. The morphological descriptions of taxa followed the methods outlined in previous studies (Parra 2013; Kerrigan 2016; Zhao et al. 2016). The voucher specimens of this study are deposited in the fungarium of the Fujian Academy of Agricultural Sciences (FFAAS), China.

### ﻿Phylogenetic studies

Genomic DNA was extracted from specimens using the DNA extraction kit (Fuzhou Meilisha Biotechnology Co., Ltd., Fuzhou, China). The primer pairs ITS1F/ITS4 (White et al. 1990; Gardes and Bruns 1993), LR0R/LR5 (Moncalvo et al. 2000) and EF1-983F/EF1-1567R (Rehner and Buckley 2005) were used to amplify the sequences of three DNA regions, ITS, nrLSU and *tef1-α*, respectively. The polymerase chain reaction (PCR) procedure was based on the protocol described by Mou and Bau (2021).

The newly generated sequences were deposited in the National Center of Biotechnology Information (NCBI) database (https://www.ncbi.nlm.nih.gov/). Sequences of phylogenetically related taxa within the same section were retrieved from NCBI and incorporated into phylogenetic analyses (Table 1). Finally, species falling within the clades of the species described in this study were selected and integrated to construct new multi-locus phylogenetic trees. *Heinemannomyces* sp. ZRL185 was used as an outgroup (Zhao et al. 2016; Ortiz-Santana et al. 2021). The ITS, nrLSU, and *tef1-α* sequences were independently aligned using the Q-INS-i algorithm via the MAFFT v.7.205 (Katoh and Standley 2013) online server (https://mafft.cbrc.jp/alignment/server/). Sequence alignments were manually adjusted in BioEdit v7.1.3.0 (Hall 1999) and subsequently concatenated for the ITS, nrLSU, and *tef1-α* regions using PhyloSuite v1.2.2 (Zhang et al. 2020). ModelFinder v2.2.0 (Kalyaanamoorthy et al. 2017) was used to select the best-fit model using BIC criterion.

**Table 1.** Sequences used in the phylogenetic analysis. Bold refers to the sequences produced from this study. “T” refers to the type specimen. “—” means no relevant genetic information.

Taxon	Voucher specimen	Country	GenBank accession numbers			References
			ITS	nrLSU	*tef1-α*	
*Agaricusandrewii*	RWK 2096	The USA	KJ877740	—	—	(Kerrigan 2016)
*A.andrewii*	RWK 1997 T	The USA	KJ877738	—	—	(Kerrigan 2016)
***A.andrewii***	**FFAAS 3387**	**China**	**PV247928**	**PV242028**	**PV261082**	**In this study**
***A.andrewii***	**FFAAS 3388**	**China**	**PV247929**	—	**PV261083**	**In this study**
***A.andrewii***	**FFAAS 3389**	**China**	**PV247930**	—	—	**In this study**
*A.arabiensis*	SQUH-DRB001	Oman	OM971854	—	—	(Hussain et al. 2022)
*A.arabiensis*	SQUH-SNT007 T	Oman	OM971855	OM971859	ON568585	(Hussain et al. 2022)
*A.argenteus*	ZRL20181598	China	MN604437	—	—	(Liu et al. 2020)
*A.argenteus*	QL20170054	China	MN604422	—	—	(Liu et al. 2020)
A.argenteussubsp.annetteae	RWK 2025	The USA	KJ877746	—	—	(Kerrigan 2016)
A.argenteussubsp.argenteus	RWK 1998	The USA	KJ877744	—	—	(Kerrigan 2016)
*A.argyropotamicus*	RWK 2017	The USA	KJ877748	—	—	(Kerrigan 2016)
*A.argyropotamicus*	F2047	France	JF727849	—	—	(Zhao et al. 2011)
*A.aristocratus*	ZRL20162182	China	MN604412	—	—	(Liu et al. 2020)
*A.aristocratus*	ZRL20162183	China	MN604413	—	—	(Liu et al. 2020)
***A.aurantifibrillosus***	**FFAAS 3390 T**	**China**	**PV247931**	**PV242029**	**PV261078**	**In this study**
***A.aurantifibrillosus***	**FFAAS 3391**	**China**	**PV247932**	**PV242030**	**PV261079**	**In this study**
***A.aurantifibrillosus***	**FFAAS 3392**	**China**	**PV247933**	**PV242031**	**PV261080**	**In this study**
*A.badiosquamulosus*	LAH35751 T	Pakistan	ON137221	OP831149	OP903342	(Bashir et al. 2024)
*A.badiosquamulosus*	LAH35752	Pakistan	ON137222	OP831150	OP903343	(Bashir et al. 2024)
*A.baiyunensis*	GDGM 87953 T	China	ON075801	ON140627	ON122987	(Liang et al. 2024)
*A.braendlei*	MO479453	The USA	OP470057	—	—	—
*A.campestris*	LAPAG370 T	Spain	KM657927	KR006607	KR006636	(Zhou et al. 2016)
*A.carassaii*	AH56324 T	Italy	NR_182917	—	—	(Bashir et al. 2024)
A.cf.altipes	RWK 1976	The USA	KJ877750	—	—	(Kerrigan 2016)
A.cf.altipes	RWK 1977	The USA	KJ877751	—	—	(Kerrigan 2016)
*A.colpeteii*	TL2424 T	Australia	JX984565	—	—	(Bashir et al. 2024)
*A.cupreobrunneus*	CA 87	France	DQ182532	—	—	(Liu et al. 2020)
*A.cupreobrunneus*	LAPAG322	Spain	JQ824136	—	—	(Liu et al. 2020)
*A.dunensis*	LAH35747 T	Pakistan	ON137217	OP835847	OP903344	(Bashir et al. 2024)
*A.dunensis*	LAH36807	Pakistan	ON158596	OP835848	OP903345	(Bashir et al. 2024)
***A.fafuinus***	**FFAAS 3393 T**	**China**	**PV247934**	**PV242032**	**PV261084**	**In this study**
***A.fafuinus***	**FFAAS 3394**	**China**	**PV247935**	**PV242033**	**PV261085**	**In this study**
*A.gastronevadensis*	SFSU DM Reno T	The USA	NR144997	—	—	(Liu et al. 2020)
*A.griseicephalus*	SFSU F-021060 T	The USA	NR144998	—	—	(Liu et al. 2020)
*A.griseicephalus*	ZRL20150352	China	MN604416	—	—	(Liu et al. 2020)
*A.iesu-et-marthae*	LAPAG41	Spain	KF447904	—	—	(Bashir et al. 2024)
*A.incultorum*	RWK 2109	The USA	KJ877766	—	—	(Kerrigan 2016)
*A.indicus*	TBGT16128 T	India	OR661746	—	—	(Arya and Pradeep 2024)
*A.indicus*	TBGT15735	India	OR661749	—	—	(Arya and Pradeep 2024)
*A.lannaensis*	SDBR-NK0564 T	Thailand	MW255657	MW255674	MW264834	(Jaichaliaw et al. 2021)
*A.lannaensis*	SDBR-NK0584	Thailand	MW255738	MW262926	MW264835	(Jaichaliaw et al. 2021)
*A.malakandensis*	ViL-60 T	Pakistan	OQ845443	OQ845442	OR296711	(Nawaz et al. 2024)
*A.malakandensis*	ViL-68	Pakistan	OQ845480	OQ845500	—	(Nawaz et al. 2024)
*A.malakandensis*	LD2012162	Thailand	KT951337	KT951493	KT951563	(Zhao et al. 2016)
*A.minipurpureus*	ZRL2010058 T	China	KX657043	KX656953	KX684947	(He et al. 2018b)
*A.minipurpureus*	ZRL2013342	China	KX657008	KX656944	KX684977	(He et al. 2018b)
*A.moellerianus*	GQ 1	The USA	KJ877767	—	—	(Kerrigan 2016)
*A.palodensis*	TBGT17483 T	India	OR661748	—	—	(Arya and Pradeep 2024)
*A.palodensis*	TBGT18550	India	OR661747	—	—	(Arya and Pradeep 2024)
*A.parviumbrus*	MEL:2382858 T	Australia	KP012732	—	—	(Bashir et al. 2024)
A.porphyrocephalussubsp.alpinus	AH47618 T	Italy	MK511988	—	—	(Parra et al. 2018b)
A.porphyrocephalussubsp.alpinus	LAPAG1030	Italy	MK511989	—	—	(Parra et al. 2018b)
A.porphyrocephalussubsp.pallidus	SFSU F-020927 T	The USA	NR145000	—	—	(Liu et al. 2020)
A.porphyrocephalussubsp.pallidus	RWK 2211	The USA	KJ877771	—	—	(Liu et al. 2020)
A.porphyrocephalussubsp.porphyrocephalus	JM 1	The USA	KJ877768	—	—	(Liu et al. 2020)
A.porphyrocephalussubsp.porphyrocephalus	RWK 2088	The USA	KJ877769	—	—	(Liu et al. 2020)
***A.shenzhenensis***	**FFAAS 3397 T**	**China**	**PV247938**	**PV242035**	**PV261076**	**In this study**
***A.shenzhenensis***	**FFAAS 3398**	**China**	**PV247939**	**PV242036**	**PV261077**	**In this study**
*A.thailandensis*	SDBR-CJ0118 T	Thailand	MW255675	MW255677	MW264832	(Jaichaliaw et al. 2021)
*A.violaceopunctatus*	LAH35767 T	Pakistan	ON158593	OP835850	—	(Bashir et al. 2024)
*A.violaceopunctatus*	LAH21719	Pakistan	ON158595	—	OP903347	(Bashir et al. 2024)
*A.wayanadensis*	TBGT18860 T	India	OR661750	—	—	(Arya and Pradeep 2024)
*A.wayanadensis*	TBGT18790	India	OR661751	—	—	(Arya and Pradeep 2024)
*A.zhangyensis*	QL20170111 T	China	MN604426	—	—	(Liu et al. 2020)
*A.zhangyensis*	QL201701152	China	MN604427	—	—	(Liu et al. 2020)
***Agaricus* sp.**	**FFAAS 3399**	**China**	**PV247940**	**PV242037**	**PV261081**	**In this study**
*Agaricus* sp.	ZRL20162141	China	MN604414	—	—	(Liu et al. 2020)
*Agaricus* sp.	RWK 1923	The USA	KJ877772	—	—	(Liu et al. 2020)
*Heinemannomyces* sp.	ZRL185	Thailand	KT951346	KT951527	KT951657	(Zhao et al. 2016)

Maximum likelihood (ML) phylogenies were inferred using IQ-TREE for 5000 ultrafast bootstraps, as well as the Shimodaira–Hasegawa–like approximate likelihood-ratio test (Guindon et al. 2010; Nguyen et al. 2015). Bayesian Inference (BI) phylogenies were inferred using MrBayes 3.2.6 (Ronquist et al. 2012) under partition model, in which the initial 25% of sampled data were discarded as burn-in. Analyses were run until convergence criteria were met (average standard deviation of split frequencies <0.01). The phylogenetic tree was visualized using FigTree v1.4.3 (http://tree.bio.ed.ac.uk/software/figtree/) and edited using Adobe Illustrator 2021 (Adobe, San Jose, CA, USA). Bootstrap support (BS) values ≥ 50% and Bayesian posterior probability (PP) values ≥ 0.70 are indicated on branches (BS/PP).

## ﻿Results

### ﻿Phylogenetic analyses

In this study, 30 sequences were generated including 11 ITS sequences, 9 nrLSU sequences, and 10 *tef1-α* sequences. The multi-locus dataset (ITS + nrLSU+ *tef1-α*) of *Agaricus* had an aligned length of 2157 bp (ITS subset: 1–725 bp; nrLSU subset:726–1595 bp; *tef1-α* subset: 1596–2157 bp) total characters including gaps. Alignment has 70 sequences with 2157 columns, 637 distinct patterns 407 parsimony-informative, 155 singleton sites, 1595 constant sites. The alignment was submitted to Figshare (https://figshare.com/s/778e265dd87f6d6f83b0).

**Figure 1.**
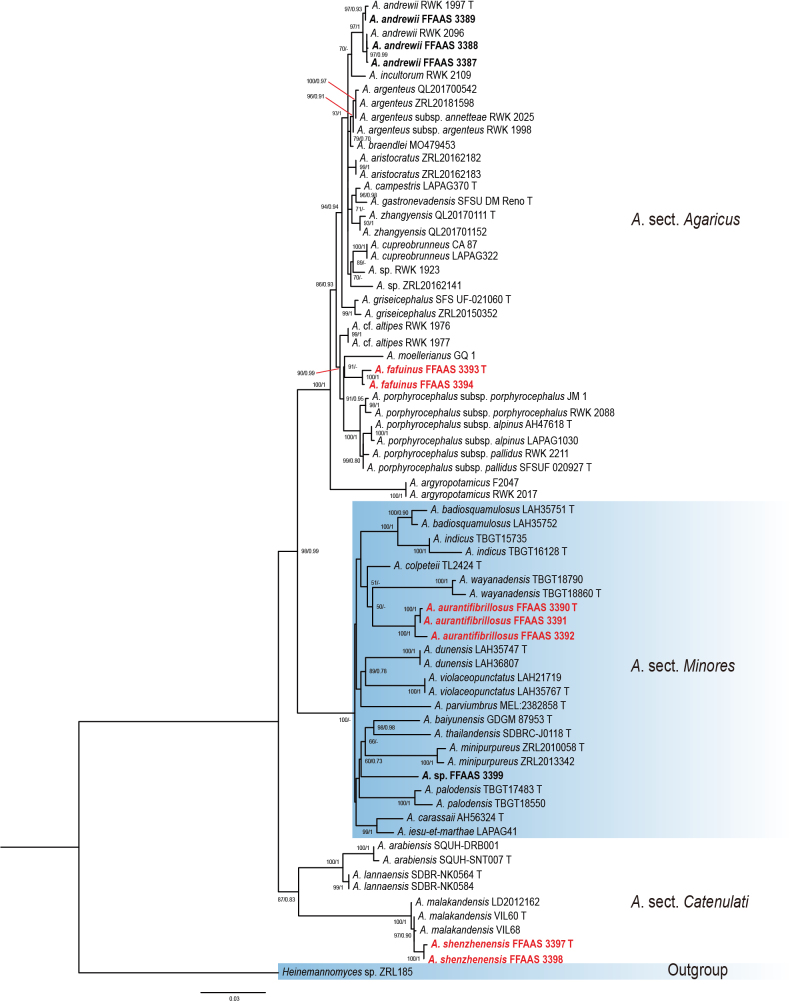
Multi-locus phylogenetic tree of *Agaricus* obtained from the maximum likelihood analysis (ML) based on ITS, nrLSU, and *tef1-α* sequence data. “T” refers to the type specimen. Bold refers to the sequences produced from this study. Red font refers to the new species.

In this study, Bayesian inference (BI) and maximum likelihood (ML) phylogenetic trees were reconstructed using a concatenated dataset comprising ITS, nrLSU, and *tef1-α* sequences. BI and ML analysis resulted in a very similar topology, so the ML tree is provided in this study (Fig. 1). For the construction of the maximum likelihood (ML) phylogenetic tree, the best-fit substitution model (TIM2+F+I+G4, without partitioning) was selected using BIC criterion. Similarly, for the Bayesian inference (BI) phylogeny, a partitioned model (edge-linked) was optimized using BIC, with the following partitions: HKY+F+G4 for ITS, GTR+F+I+G4 for nrLSU, and K2P+G4 for *tef1-α*.

The phylogenetic tree revealed three major clades, corresponding to three sections: A.sect.Agaricus, A.sect.Catenulati and A.sect.Minores. *A.fafuinus* and *A.moellerianus* Bon formed a sister clade (BS/PP = 91/-) in A.sect.Agaricus. Within A.sect.Catenulati, *A.shenzhenensis* and *A.malakandensis* formed a sister clade with a strong support value (BS/PP = 97/0.90). Within A.sect.Minores, *A.aurantifibrillosus* is phylogenetically close to *A.colpeteii* T. Lebel and *A.wayanadensis*. Despite low support values, *A.aurantifibrillosus* forms a distinct clade.

### ﻿Taxonomy

#### ﻿ Agaricus aurantifibrillosus

Taxon classificationFungiAgaricalesAgaricaceae

P.H. Liu & S.E. Wang sp. nov.

E376667B-C689-5DE7-95D9-EF79C43A28CC

858060

Figs 2A, B , 3

##### Etymology.

*aurantifibrillosus* (Latin), referring to the pileus covered with arranged orange (5A8) or brownish yellow (5C8) fibrillose squamules.

**Figure 2.**
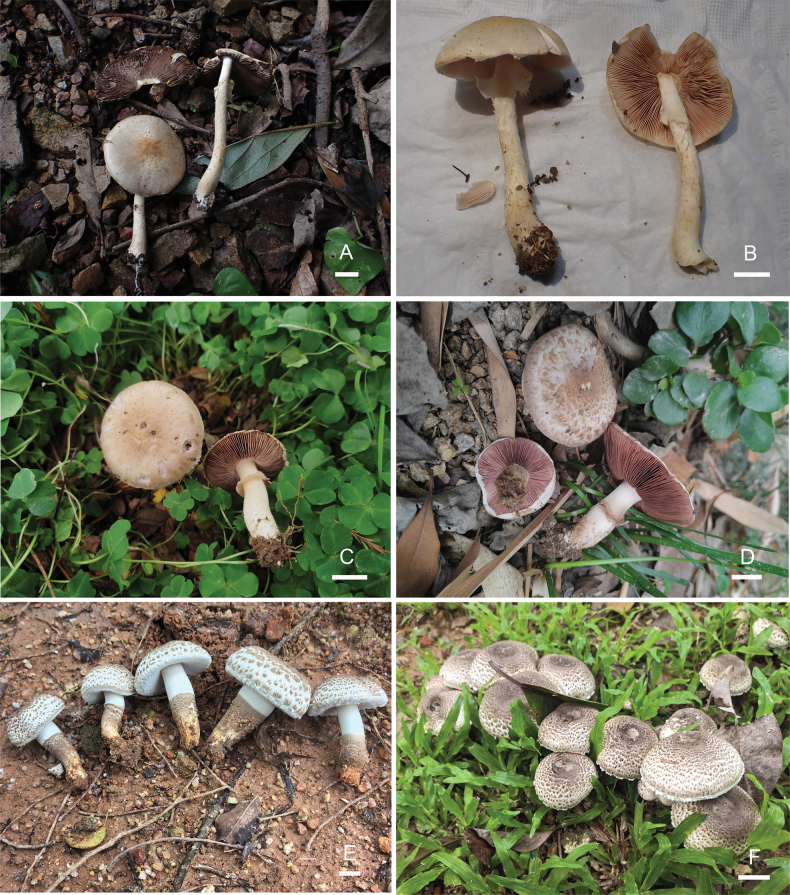
The photographs of fresh basidiomata of *Agaricus* species in this study. **A.***A.aurantifibrillosus* (FFAAS 3390); **B.***A.aurantifibrillosus* (FFAAS 3391); **C.***A.fafuinus* (FFAAS 3393); **D.***A.fafuinus* (FFAAS 3394); **E.***A.shenzhenensis* (FFAAS 3397); **F.***A.shenzhenensis* (FFAAS 3398). Scale bars: 1 cm.

##### Holotypus.

China • Fujian Province, Fuzhou City, Fuzhou National Forest Park, 2 October 2024, 26°10'41"N, 119°16'19"E, alt. 280 m, Shi-En Wang, E2410232 (FFAAS 3390).

**Figure 3.**
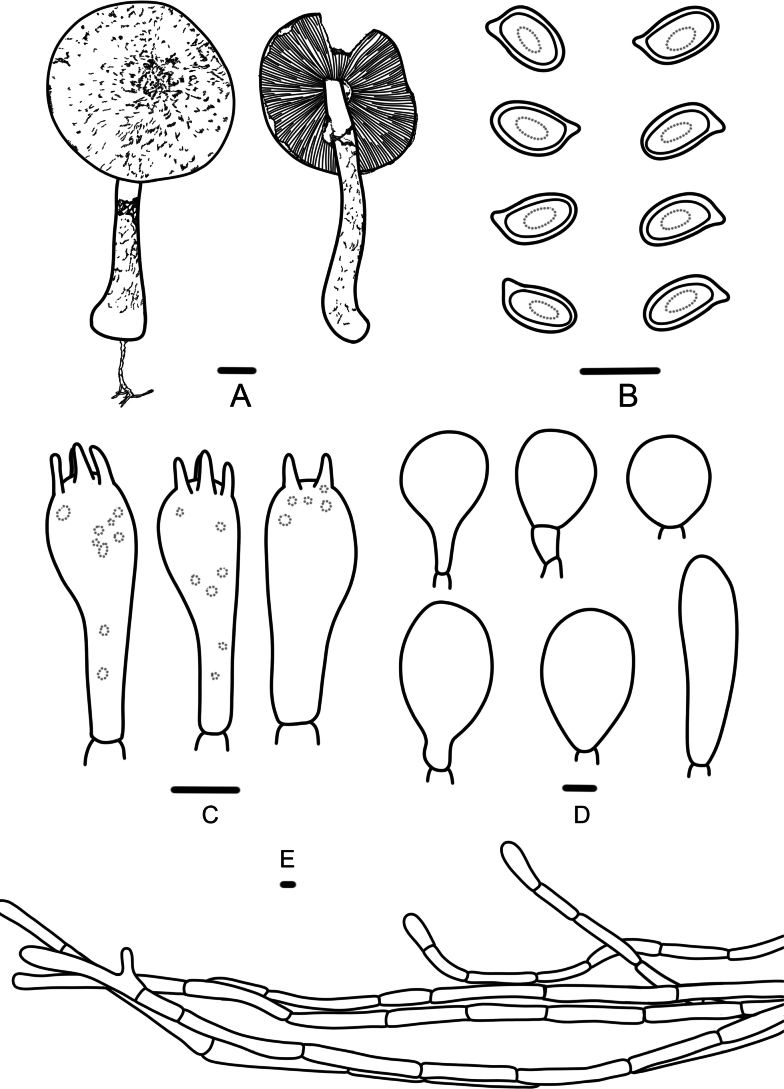
*Agaricusaurantifibrillosus* (FFAAS 3390, FFAAS 3391); **A.** Basidiomata; **B.** Basidiospores; **C.** Basidia; **D.** Cheilocystidia; **E.** Pileipellis. Scale bar: 1 cm (**A**); 5 μm (**B–E**).

##### Diagnosis.

This species is characterized by its pileus covered with orange (5A8) or brownish yellow (5C8) scattered fibrillose squamules, elongate basidiospores (Qav = 1.70), and abundant cheilocystidia.

##### Description.

Pileus 3.6–6.2 cm in diameter, 0.2–0.3 cm thick at the center, truncate conical to plane, surface dry, white (5A1) or orange white (5A2), covered with scattered arranged orange (5A8), brownish yellow (5C8) fibrillose squamules, denser at disc, showing brownish orange (6C8) or brown (6E8), margin appendiculate by annulus remnants. Context of the pileus white (6A1), with no special odor. Lamellae 0.2–0.4 cm broad, pastel red (9A4) then reddish brown (8E8) later brownish black (8F8), free, crowded, intercalated with numerous lamellulae. Stipe 5.6–8.6 × 0.4–1.3 cm, hollow, clavate, with white (6A1) rhizomorphs, provided with an annulus in its upper third, above the annulus white (6A1), below the annulus with white (6A1), orange (5A8) floccose squamules, becoming dark yellow (4C8) on touching or bruising. Annulus superior, white (6A1), simple, membranous, easy falling out.

Basidiospores (4.6)4.7–5.7(5.9) × (2.7)2.8–3.3(3.6) μm, [Xav = 5.2 × 3.1 μm], Q = 1.50–1.93, Qav = 1.70, ellipsoid to elongate-ellipsoid, smooth, thick-walled, brown, guttulate. Basidia 14–18 × 5–7 μm, clavate, 4(2)-spored, sterigmata 2–4 µm long. Cheilocystidia abundant, nearly globose, oblong, sphaeropedunculate, or broadly clavate, 12–34 × 10–20 μm, with pale yellowish intracellular pigment. Pleurocystidia absent. Pileipellis a cutis of cylindrical, slightly constricted at the septa, pale yellowish hyphae, 4–9 μm in diameter.

##### Habitat and distribution.

Gregarious or scattered in broad-leaved and bamboo forests during autumn. Currently, it has only been documented in Fujian Province, China.

##### Additional specimens measured.

China • Fujian Province, Fuzhou City, Fujian Agriculture and Forestry University, 5 October 2024, Shi-En Wang, E2410524 (FFAAS 3391) and E2410525 (FFAAS 3392).

##### Notes.

*Agaricusaurantifibrillosus* belongs to A. (subg. Minores) sect. Minores. *Agaricusaurantipileatus* T. Bau & S.E. Wang in A.sect.Arvenses shares morphological similarities with *A.aurantifibrillosus*. However, *A.aurantipileatus* can be distinguished by its double annulus, smaller spore Q value (Q = 1.17–1.36), and sometimes catenulate cheilocystidia (Wang and Bau 2024).

In the multi-locus phylogenetic tree (Fig. 1), *A.aurantifibrillosus* clusters with *A.colpeteii* T. Lebel and *A.wayanadensis*, albeit with low statistical support, and this may be due to the lack of sequences in the related taxa. *Agaricuscolpeteii* is a gasteroid *Agaricus* species with basidia not observed (Lebel 2013). *Agaricuswayanadensis* differs in having a pileus surface covered with brown squamules and a smaller spore Qav value (Qav = 1.58) (Arya and Pradeep 2024).

#### ﻿ Agaricus fafuinus

Taxon classificationFungiAgaricalesAgaricaceae

P.H. Liu & S.E. Wang sp. nov.

5F9C3FAF-FE6A-578F-A022-5F090B7BFB5A

858061

Figs 2C, D , 4

##### Etymology.

Derived from the acronym FAFU (Fujian Agriculture and Forestry University), where the type specimens of this species were collected.

**Figure 4.**
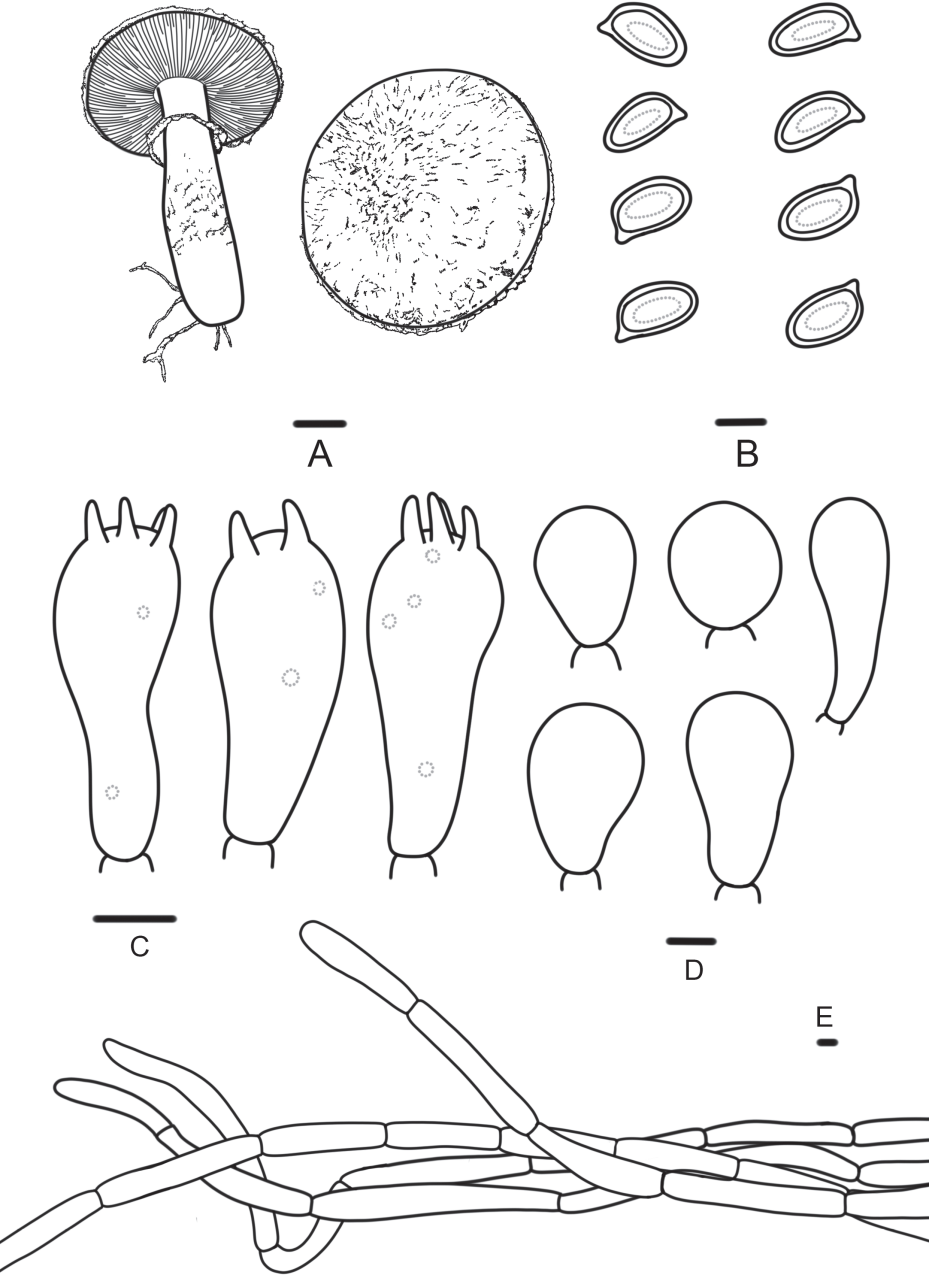
*Agaricusfafuinus* (FFAAS 3393, FFAAS 3394); **A.** Basidiomata; **B.** Basidiospores; **C.** Basidia; **D.** Cheilocystidia; **E.** Pileipellis. Scale bar: 1 cm (**A**); 5 μm (**B–E**).

##### Holotypus.

China • Fujian Province, Fuzhou City, Fujian Agriculture and Forestry University, 4 March 2024, 26°08'N, 119°24'E, alt. 30 m, Si-Ang Chen, CSA299 (FFAAS 3393).

##### Diagnosis.

This species is characterized by its pileus and stipe covered with brown (7D5) or fox red (8D7) fibrils or fibrillose squamules, elongate basidiospores (Qav = 1.63), and abundant cheilocystidia.

##### Description.

Pileus 3.4–5.5 cm in diameter, 0.2–0.4 cm thick at the center, truncate conical to plane, surface dry, white (5A1), brownish gray (7C2), covered with brown (7D5) or fox red (8D7) fibrils or with fibrillose squamules, concentrically arranged, denser and reddish brown (8E8) at disc, margin appendiculate by annulus remnants. Context of the pileus, white (6A1), with no special odor. Lamellae 0.2–0.3 cm broad, first pastel red (9A4), then reddish brown (8E8), later brownish black (8F8), free, crowded, intercalated with numerous lamellulae. Stipe 2.2–4.5 × 0.6–1.5 cm, nearly cylindrical, hollow, with white (6A1) rhizomorphs, provided with an annulus in its upper half, above the annulus white (6A1), below the annulus covered towards the base with concolorous squamules with the pileus surface. Annulus superior, white (6A1) to reddish brown (8E8), simple, membranous, persistent.

Basidiospores (5.7)6.0–7.9(8.2) × (3.5)3.7–5.0(5.1) μm, [Xav = 6.8 × 4.2 μm], Q = 1.46–2.03, Qav = 1.63, ellipsoid to cylindrical, smooth, thick-walled, brown, guttulate. Basidia 17–25 × 6–9 μm, clavate, 4(2)-spored, sterigmata 1–3 µm long. Cheilocystidia abundant, nearly globose, broadly clavate, or pyriform, 10–23 × 7–13 μm, hyaline. Pleurocystidia absent. Pileipellis a cutis of cylindrical, slightly constricted at septa, light brown hyphae, 6–12 μm wide.

##### Habitat and distribution.

Gregarious in bamboo forests or grass during spring. Currently, it has only been known from Fujian Province, China.

##### Additional specimens measured.

China • Fujian Province, Fuzhou City, Fujian Agriculture and Forestry University, 23 March 2024, Si-Ang Chen, CSA314 (FFAAS 3394).

##### Notes.

*Agaricusfafuinus* belongs to A. (subg. Agaricus) sect. Agaricus. *Agaricusfafuinus* exhibits variable pileus surface: specimen CSA299 possesses fibrils, while CSA314 displays fibrillose squamules. However, molecular data confirm their conspecificity, suggesting that these morphological differences may result from humidity variations. Specimen CSA299 was collected post-rainfall and likely it was exposed to precipitation, whereas specimen CSA314 was obtained under dry conditions. Initially, we thought we had mixed up the specimen numbers, but after thorough verification, this turned out to be accurate.

In the multi-locus phylogenetic tree (Fig. 1), *A.fafuinus* and *A.moellerianus* Bon form a sister clade with good support. However, *A.moellerianus* is distinguished by its subglabrous white pileus, smooth stipe, rounder basidiospores (Qav = 1.22), and broadly clavate cheilocystidia resembling basidioles (Kerrigan 2016).

#### ﻿ Agaricus shenzhenensis

Taxon classificationFungiAgaricalesAgaricaceae

P.H. Liu & S.E. Wang sp. nov.

9957CD28-9E14-588F-A514-BE678DB58C09

858063

Figs 2E, F , 5

##### Etymology.

*shenzhenensis* (Latin), meaning from shenzhen city where the holotype specimen was collected.

**Figure 5.**
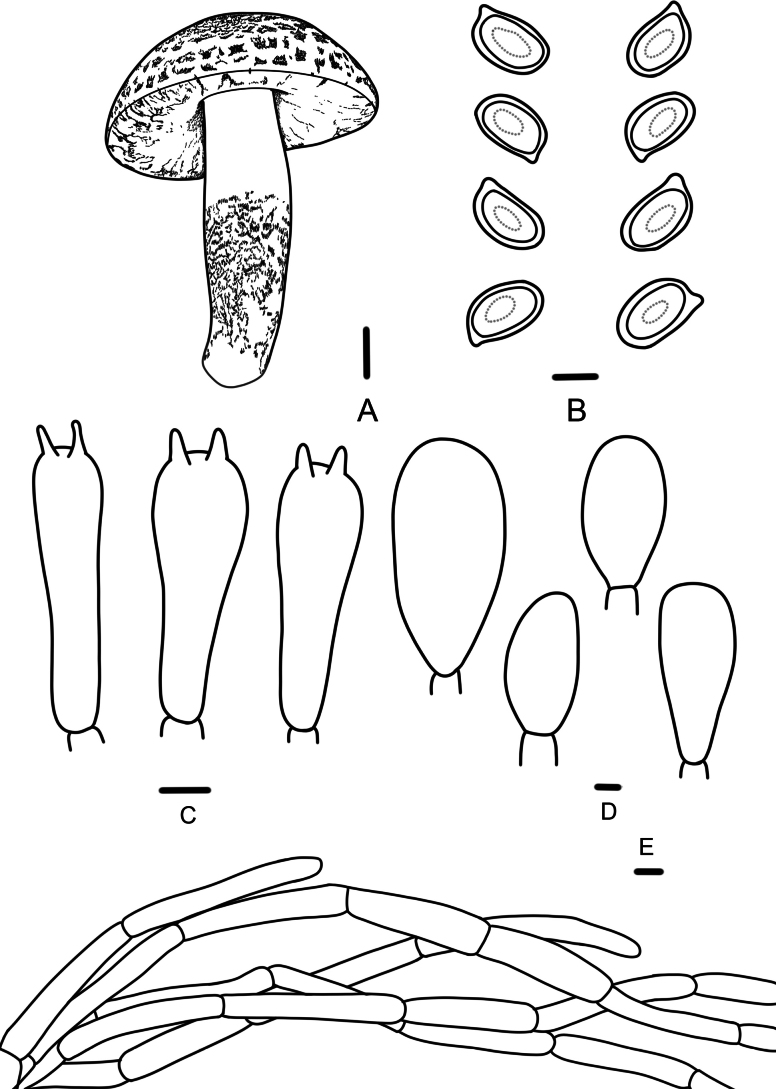
*Agaricusshenzhenensis* (FFAAS 3397, FFAAS 3398); **A.** Basidiomata; **B.** Basidiospores; **C.** Basidia; **D.** Cheilocystidia; **E.** Pileipellis. Scale bar: 1 cm (**A**); 5 μm (**B–E**).

##### Holotypus.

China • Guangdong Province, Shenzhen City, Lianhuashan Park, 29 April 2024, 22°33'23"N, 114°3'13"E, alt. 100 m, Cheng-Cheng, 20240430 (FFAAS 3397).

##### Diagnosis.

Distinguished by the pileus adorned with brownish gray (6E2) fibrillose squamules, context becoming yellow (3B8) on cutting, stipe base tapering and covered with brownish gray (6E2) fibrillose squamules, predominantly 2-spored basidia.

##### Description.

Pileus 2.5–6.5 cm in diameter, 0.5–0.8 cm thick at the center, hemispherical, truncate conical to plane, applanate with a slightly depressed center when mature, surface dry, white (2A1), grayish white (2B1), entirely covered with appressed, concentrically arranged, triangular, brownish gray (6E2) fibrillose squamules, scattered towards the margin in age, denser taupe (4F1), black (2F1) at the disc, margin entire, appendiculate by annulus remnants. Context of the pileus white (6A1), becoming yellow (3B8) on cutting, odor unknown. Lamellae 0.5–0.7 cm broad, first pale red (9A3) then reddish brown (8E8), later brownish black (8F8), free, crowded, intercalated with numerous lamellulae. Stipe 4.5–11.5 × 0.5–2.0 cm, cylindrical, tapering downwards, hollow, with short white (6A1) rhizomorphs, provided with an annulus in its upper part, above the annulus white (6A1), below the annulus the upper half white and the lower half covered with dense adpressed brownish grey (6E2) squamules concolorous with the pileus surface. Annulus superior, white (6A1) to brownish black (8F8), simple, membranous, persistent.

Basidiospores (5.8)6.1–7.3(7.5) × (3.8)4.0–4.3(4.4) μm, [Xav = 6.8 × 4.2 μm], Q = 1.41–1.87, Qav = 1.61, ellipsoid to elongate-ellipsoid, smooth, brown, thick-walled, guttulate. Basidia 25–33 × 8–10 μm, clavate, 2(4)-spored, sterigmata 2–3 µm long. Cheilocystidia abundant, broadly clavate, oblong or pyriform, 22–50 × 10–22 μm. Pleurocystidia absent. Pileipellis a cutis of cylindrical, slightly constricted at the septa, light brown hyphae, 4–7 μm wide.

##### Habitat and distribution.

Gregarious or clustered in grass or broad-leaved forests during spring. Currently, it is only known from Guangdong Province, China.

##### Additional specimens measured.

China • Guangdong Province, Shenzhen City, Lianhuashan Park, 29 April 2024, Cheng-Cheng, 20240430-1 (FFAAS 3398).

##### Notes.

*Agaricusshenzhenensis* belongs to A. (subg. Pseudochitonia) sect. Catenulati, which currently comprises three species: *A.arabiensis* S. Hussain & Al-Sadi, *A.lannaensis* N. Suwannarach, J. Kumla & S. Lumyong and *A.malakandensis*.

*Agaricusshenzhenensis* differs from all three in both morphological and molecular characters. *Agaricusarabiensis* exhibits a reddish-brown to dark reddish-brown pileus, context unchanged on handling, smooth stipe, and globose to subglobose or broadly clavate, regularly catenulate cheilocystidia (Hussain et al. 2022). *Agaricuslannaensis* differs in having brown pileus, reddish brown context when cut, fibrillose stipe white below the annulus to the base, and smaller basidia (19–26 × 5.5–8.5 µm) (Jaichaliaw et al. 2021). *Agaricusmalakandensis* possesses a dark brown to reddish brown pileus, smooth or fibrillose to slightly squamulose stipe, and multiseptate cheilocystidia with clavate to pyriform terminal element (Nawaz et al. 2024). Notably, catenulate cheilocystidia were not observed in this species, even though this feature is the primary morphological diagnostic characteristic for taxa within this section.

The genetic distinctions between *A.shenzhenensis* and *A.malakandensis* remain unequivocal, with four nucleotide differences in the ITS region, one in nrLSU, and four in *tef1-α* (Table 2). To ensure the accuracy of the results, we rechecked the quality of the sequences and confirmed their compliance with the required standards.

**Table 2.** Variable loci of *A.malakandensis* and *A.shenzhenensis*. Position numbering based on ITS/nrLSU/*tef1-α* alignment. “—” means no relevant genetic information.

Sample	ITS				nrLSU	*tef1-α*			
	256	273	286	646	410	288	399	439	503
*A malakandensis*									
ViL-60 T	C	T	C	C	C	G	A	C	C
ViL-68	C	T	C	C	C	—	—	—	—
LD2012162	C	T	C	C	C	G	A	C	C
*A shenzhenensis*									
FFAAS 3397 T	T	C	T	T	G	A	T	T	T
FFAAS 3398	T	C	T	T	G	A	T	T	T

Notably, this species represents the first record of A.sect.Catenulati in China, expanding the known biogeographic range of this section.

## ﻿Discussion

Of the 26 recently described *Agaricus* species, five, *A.calolepidotus*, *A.xalapensis*, *A.karakensis*, *A.palodensis* and *A.wayanadensis*, were delineated solely through ITS sequence data (Arya and Pradeep 2024; Guzmán-Guillermo et al. 2024; Ullah et al. 2024), without multi-locus phylogenetic analyses. The same applies to *A.totalaiiensis*, which is currently under review. Notably, the original description of *A.karakensis* lacks explicit GenBank accession numbers for its molecular data (Ullah et al. 2024). However, phylogenetic analyses of *Agaricus* based exclusively on ITS sequences lack methodological rigor (Cao et al. 2020). To ensure taxonomic reliability, the description of novel *Agaricus* species should integrate analyses of multi-locus (e.g., ITS + nrLSU + *tef1-α*). Furthermore, resolving higher-level phylogenetic relationships using single-copy orthologous genes derived from genomic data (He et al. 2024; Wang et al. 2024) is emerging as a trend.

Convergent evolution is a notable phenomenon within *Agaricus*, exemplified by striking macro-morphological similarities between *A.aurantifibrillosus* and *A.aurantipileatus*. Remarkably, this convergence extends beyond intra-generic boundaries, as seen in *A.sinoagrocyboides* T. Bau & S.E. Wang, a species our team previously discovered in China, which exhibits macroscopic traits strongly resembling those of *Agrocybe* species (Wang and Bau 2024). Such morphological variability becomes more complex in certain identifications due to the ecological plasticity of *Agaricus*. For instance, the fact that *A.subiculosus* is growing on decaying wood challenges traditional habitat assumptions for *Agaricus* (Parra et al. 2024a). These observations underscore the necessity of collecting morphologically ambiguous specimens during field surveys, as overlooked specimens may represent cryptic lineages or novel ecological adaptations within *Agaricus*.

During our field surveys targeting *Agaricus* specimens, we inadvertently collected specimens of the genera *Leucoagaricus*, *Leucocoprinus*, *Micropsalliota* and *Xanthagaricus*. These specimens will undergo comprehensive morphological and molecular analyses to explore potential novel discoveries.

## Supplementary Material

XML Treatment for Agaricus aurantifibrillosus

XML Treatment for Agaricus fafuinus

XML Treatment for Agaricus shenzhenensis

## ﻿Additional information

### Author ORCIDs

Shi-en Wang https://orcid.org/0009-0002-0710-0728

Si-ang Chen https://orcid.org/0009-0007-7315-8107

### Data availability

All of the data that support the findings of this study are available in the main text, or in publicly accessible data repositories, as indicated in the text.
